# Feasibility of four-dimensional preoperative simulation for elbow debridement arthroplasty

**DOI:** 10.1186/s12891-016-0996-9

**Published:** 2016-04-02

**Authors:** Michiro Yamamoto, Yukimi Murakami, Katsuyuki Iwatsuki, Shigeru Kurimoto, Hitoshi Hirata

**Affiliations:** Department of Hand Surgery, Nagoya University Graduate School of Medicine, 65 Tsurumai-cho, Showa-ku, Nagoya 466-8550 Japan

**Keywords:** Four-dimensional simulation, Debridement arthroplasty, Elbow osteoarthritis

## Abstract

**Background:**

Recent advances in imaging modalities have enabled three-dimensional preoperative simulation. A four-dimensional preoperative simulation system would be useful for debridement arthroplasty of primary degenerative elbow osteoarthritis because it would be able to detect the impingement lesions.

**Methods:**

We developed a four-dimensional simulation system by adding the anatomical axis to the three-dimensional computed tomography scan data of the affected arm in one position. Eleven patients with primary degenerative elbow osteoarthritis were included. A “two rings” method was used to calculate the flexion-extension axis of the elbow by converting the surface of the trochlea and capitellum into two rings. A four-dimensional simulation movie was created and showed the optimal range of motion and the impingement area requiring excision. To evaluate the reliability of the flexion-extension axis, interobserver and intraobserver reliabilities regarding the assessment of bony overlap volumes were calculated twice for each patient by two authors. Patients were treated by open or arthroscopic debridement arthroplasties. Pre- and postoperative examinations included elbow range of motion measurement, and completion of the patient-rated questionnaire Hand20, Japanese Orthopaedic Association-Japan Elbow Society Elbow Function Score, and the Mayo Elbow Performance Score.

**Results:**

Measurement of the bony overlap volume showed an intraobserver intraclass correlation coefficient of 0.93 and 0.90, and an interobserver intraclass correlation coefficient of 0.94. The mean elbow flexion-extension arc significantly improved from 101° to 125°. The mean Hand20 score significantly improved from 52 to 22. The mean Japanese Orthopaedic Association-Japan Elbow Society Elbow Function Score significantly improved from 67 to 88. The mean Mayo Elbow Performance Score significantly improved from 71 to 91 at the final follow-up evaluation.

**Conclusion:**

We showed that four-dimensional, preoperative simulation can be generated by adding the rotation axis to the one-position, three-dimensional computed tomography image of the affected arm. This method is feasible for elbow debridement arthroplasty.

**Electronic supplementary material:**

The online version of this article (doi:10.1186/s12891-016-0996-9) contains supplementary material, which is available to authorized users.

## Background

Primary degenerative elbow osteoarthritis causes the loss of range of motion and pain at the end of motion. Some patients may benefit from removal of loose bodies or debridement with removal of prominent osteophytes [1]. Several techniques reported for debridement arthroplasty for elbow osteoarthritis include a posterolateral approach, lateral and medial approach, posteromedial approach, medial trans-flexor approach, and arthroscopic approach [2–7]. Although each technique has advantages and disadvantages, it is challenging for the surgeon to identify the critical impingement area in the complicated degenerated elbow.

Recent advances in imaging modalities have enabled four-dimensional preoperative simulation. In the last decade, many studies have investigated four-dimensional computed tomography (CT) [8, 9]. The four dimensions considered in this method include the three spatial dimensions and time [10]. By calculating the joint axis to move the elbow joint and adding a time dimension to three-dimensional CT images, we can create a four-dimensional simulation for debridement arthroplasty for elbow osteoarthritis using computer-aided design (CAD) software. The four-dimensional simulation can help surgeons identify impingement lesions that should be removed.

The position and orientation of the elbow flexion axis vary in vivo, indicating that the elbow flexion-extension axis is not a line, but varies throughout the arc of motion [11]. However, the mean axes are located close to a line joining the centers of the trochlea and capitellum [12, 13]. A “two rings” method was used to calculate the flexion-extension axis by converting the surface of the trochlea and capitellum of the humerus into two rings. To create the two rings, any three points were plotted on both the trochlea and capitellum of the humerus. Then, two rings were drawn through the closest of the three points. The flexion-extension axis can be drawn through the centers of two rings. Therefore, elbow flexion and extension can be simulated by rotating the radius and ulna around the calculated axis. If the axis is far from optimal, we can easily detect the error by watching the four-dimensional simulation movie (Additional file 1).

The purpose of this study was to develop a four-dimensional preoperative simulation system by adding the anatomical axis to the three-dimensional CT scan data obtained with the affected arm in one position and to determine the feasibility of the simulation in the clinical setting. We hypothesized that the “two rings” method is useful for the identification of the elbow flexion-extension axis.

## Methods

### Patients

Between February 2012 and August 2013, 11 patients with impingement pain at the extremes of motion due to moderate or severe primary degenerative elbow osteoarthritis, with or without cubital tunnel syndrome, were included in this study. Institutional review board approval of Nagoya University Graduate School of Medicine (2011–0023) was granted before initiation of the study, and the patients gave their written informed consent for participation. The study included 9 men and 2 women with a mean age of 60 years (range 48 to 72 years) at the time of surgery. All patients had symptoms associated with limited elbow flexion-extension (Table 1).

**Table 1:** Demographic data of all patients

Case	Sex	Age	Flexion (pre-op)	Extension (pre-op)	Arc (pre-op)	CuTS	Surgery	Flexion (post-op)	Extension (post-op)	Arc (post-op)
1	F	68	100	−30	70	-	Scopic	140	0	140
2	M	65	108	−45	63	+	Open	130	−15	115
3	M	52	125	−5	120	-	Scopic	130	0	130
4	F	68	130	−20	110	+	Open	140	−10	130
5	M	48	130	−10	120	-	Scopic	140	0	140
6	M	56	110	−25	85	+	Open	130	−10	120
7	M	72	115	−20	95	+	Open	123	−22	101
8	M	60	110	0	110	+	Open	118	0	118
9	M	69	130	−10	120	-	Scopic	134	−10	124
10	M	50	130	−10	120	-	Scopic	135	−5	130
11	M	51	110	−15	95	+	Open	125	−10	115

*CuTS* cubital tunnel syndrome

### Preoperative four-dimensional simulation technique

**Fig. 1:**
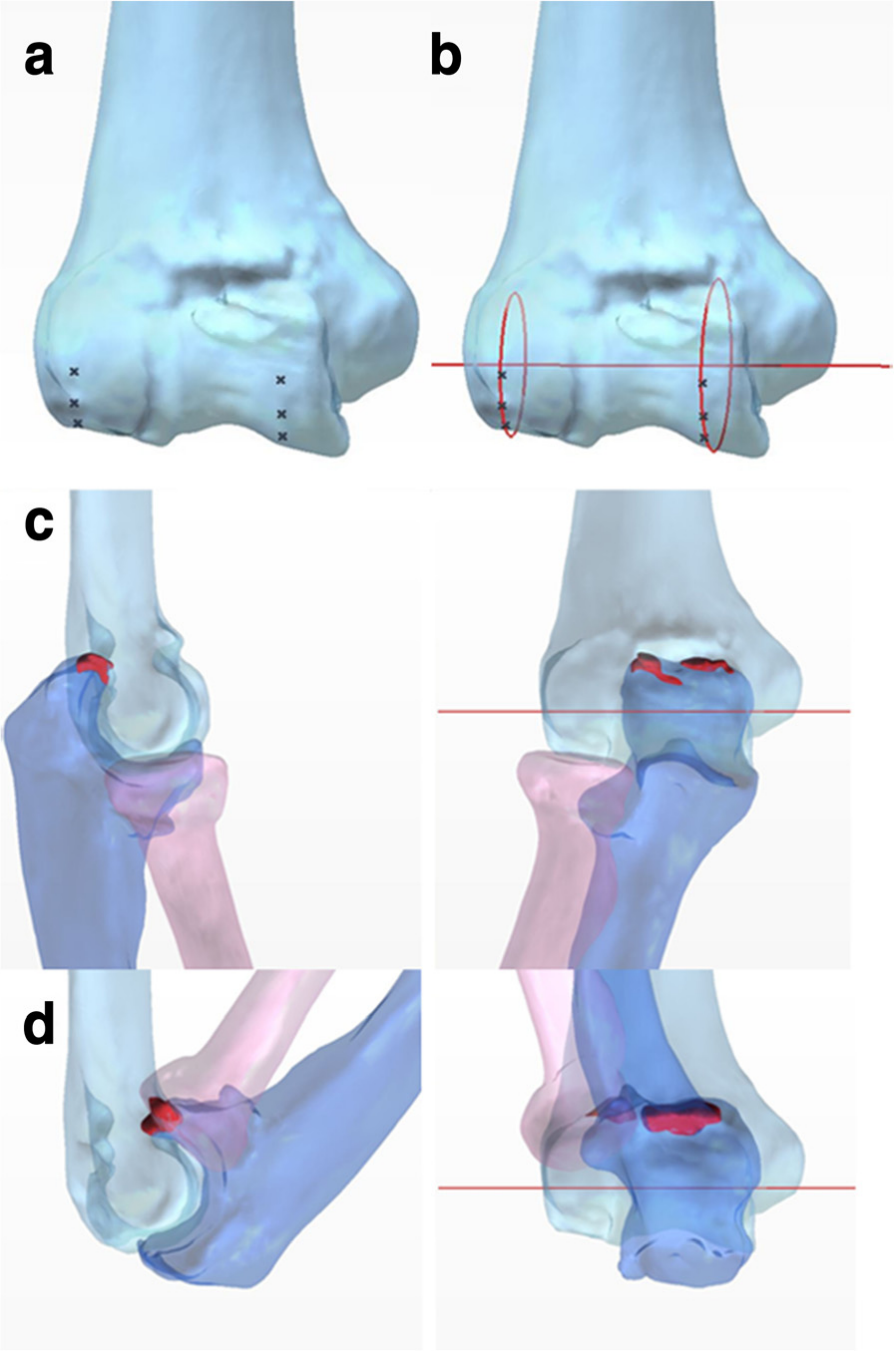
Overview of the “two rings” method (case 11). Any three points were plotted on the trochlea and capitellum of the humerus (**a**). Two rings are drawn through the closest of the three points. The flexion-extension axis passes through the centers of the two rings (**b**). The optimal range of motion and the impingement area (red lesion) are shown (**c** elbow flexion 0°, **d** 140°)

CT scan images of the affected elbow in extension were obtained once preoperatively. CT scans were stored in stereolithography (STL) format for subsequent processing using Mimics software (Materialize, Leuven, Belgium). Three-dimensional solid models were created from CT scans using ThinkDesign software (think3, Bologna, Italy). The elbow flexion-extension axis was calculated using the “two rings” method in the distal humerus. The “two rings” method was used to calculate the flexion-extension axis of the elbow by converting the surface of the trochlea and capitellum of the humerus into two rings (Fig. 1a and b). First, any three points were plotted on both the trochlea and capitellum of the humerus. When selecting these points, we usually avoided irregular areas of the trochlea and capitellum that had bony spurs and/or erosion. Then, two rings were drawn through the closest of the three points. The mean flexion-extension axes are located close to a line joining the centers of the trochlea and capitellum [14]. Therefore, the axis passes through the centers of two rings. Preoperative four-dimensional simulation was completed with the axis. The axis was examined in terms of the impingement and alignment to confirm the absence of any inconsistency with the clinical information regarding the range of motion. A four-dimensional simulation movie was created (Additional file 1); this movie showed the optimal range of motion (ROM) and impingement area requiring excision (Fig. 1c and d).

To evaluate the reliability of the flexion-extension axis, interobserver and intraobserver reliabilities regarding the assessment of bony overlap volumes were calculated twice for each patient by two authors (YM and MY). The impingement area was computed at elbow flexion angles of 0° and 140° because achieving these degrees of flexion is the usual goal of both patients and surgeons after debridement arthroplasty [7]. Intraobserver and interobserver reliabilities were assessed using one-way random intraclass correlation coefficient (ICC) and two-way random ICC, respectively. The reliability is considered excellent if the ICC is >0.75, fair to good if 0.4 < ICC < 0.75, and poor if ICC is <0.4 [15]. All data were analyzed using IBM SPSS Statistics for Windows, Version 20.0 (IBM Corp., Armonk, NY, USA).

### Surgical technique

Surgeons and the other operating staff are able to watch the four-dimensional simulation movie preoperatively and intraoperatively by bringing a personal computer into the operating theater.

Patients with cubital tunnel syndrome were treated by open debridement arthroplasty after ulnar neurolysis. Patients without cubital tunnel syndrome were treated by open or arthroscopic debridement arthroplasties according to the surgeon’s preference.

Five patients without cubital tunnel syndrome were treated arthroscopically, and six patients with cubital tunnel syndrome underwent open surgery.

Open debridement arthroplasty was performed under general anesthesia or axillary block, and the patient was placed in the supine position. A tourniquet was applied to the upper arm. A medial approach was used, and the ulnar nerve was released in all patients [4, 5]. Joint debridement and removal of impingement osteophytes were performed according to the preoperative four-dimensional simulation. We removed osteophytes from the coronoid, coronoid fossa, tip and sides of the olecranon, and olecranon fossa. Capsular release was also performed if necessary, regardless of the simulation. If there was an impingement lesion in the radiocapitellar joint, the lateral compartment was exposed through a lateral approach. Osteophytes were removed from the radial fossa and the posterior edge of the capitellum.

Debridement arthroplasty was performed until the appropriate elbow ROM was achieved. Simple neurolysis of the ulnar nerve was performed in four patients who underwent open surgery. The ulnar nerve was transposed subcutaneously in two cases, according to the surgeon’s preference. The wound was closed over a suction drain in the joint.

The surgical technique for arthroscopic debridement arthroplasty involved the standard arthroscopic technique. General anesthesia was induced, and the patient was placed in the lateral decubitus position. The anteromedial portal was established first, with care taken to avoid ulnar nerve injury. The anterolateral portal was established using an inside-out technique. A 3.5-mm arthroscopic shaver was introduced through the anterolateral portal. Loose bodies were removed if present. Osteophytes in the coronoid and radial head fossa were identified and removed with a shaver and burr. After completion of the procedure on the anterior aspect of the joint, the posterior joint was visualized. The posterolateral portal was established on the soft spot, and a shaver was placed in the direct posterior portal. Osteophytes were removed from the tip and sides of the olecranon and fossa [6]. After completion of the procedure, the portals were closed with 4-0 nylon suture.

### Postoperative management

No splint was applied after either open or arthroscopic debridement arthroplasty. Patients were allowed to use the affected hand on postoperative day 1, and active ROM exercises were performed under the supervision of a hand therapist.

### Assessment

ROM of the elbow, the patient-rated questionnaire Hand20, Japanese Orthopaedic Association-Japan Elbow Society Elbow Function Score (JOA-JES score), the Mayo Elbow Performance Score (MEPS) and a numeric rating scale for pain were assessed preoperatively and at postoperative final follow-up [16, 17]. Patients were divided into two groups —open or arthroscopic surgery—and compared.

We classified complications into grade I: minor complication without any unexpected surgery or anesthesia, and grade II: major complication with unexpected surgery or anesthesia.

### Statistical analysis

Data analysis was performed using Student *t*-test. A value of *P* < 0.05 was considered significant.

## Results

**Fig. 2:**
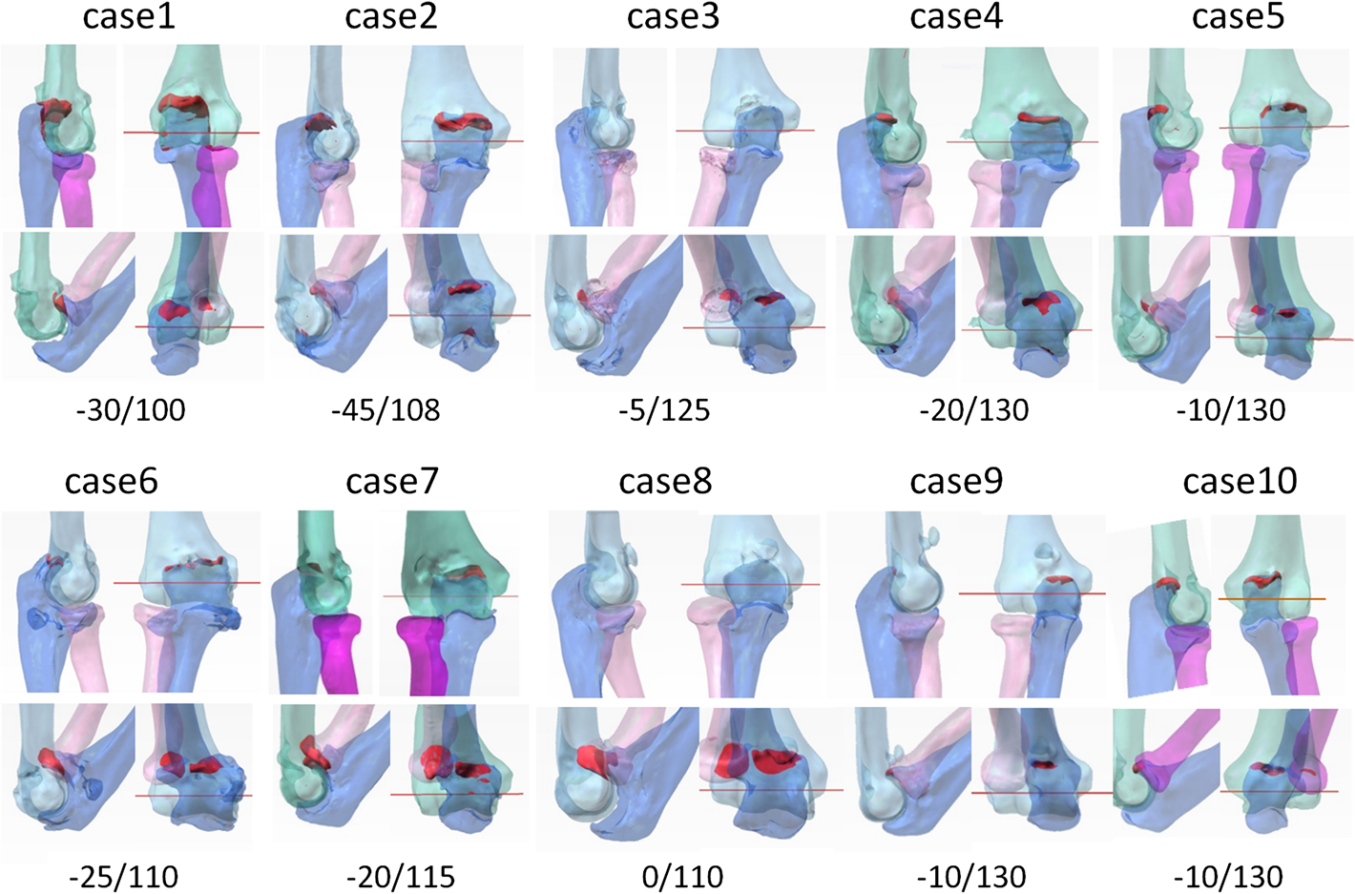
Preoperative computer simulation in all patients. Preoperative computer simulations in all patients are shown (from case 1 to 10). The result of case 11 is shown in Fig. 1. The impingement area was computed at elbow flexion angles of 0° and 140°, and shown in red. Preoperative clinical data of extension and flexion angles are shown below

All preoperative computer simulations at elbow flexion angles of 0° and 140° are shown in Fig. 2. The impingement area was computed and shown in red. These results were consistent with the clinical findings. The impingement area appeared during simulation beyond the preoperative range of motion (Fig. 2).

Preoperative simulation was useful to help surgeons decide whether a lateral incision was necessary, and where to make an appropriate portal for the arthroscopic shaver. The surgeon could easily identify the bony impingement lesion in the complicated degenerated elbow by watching the four-dimensional simulation movie pre- and intraoperatively. In the open surgery group, four (cases 6, 7, 8 and 11) were treated using both a medial and lateral approach and two (cases 2 and 4) were treated using a medial approach. In the arthroscopic surgery group, the surgeon could treat the impingement lesion intensively. The follow-up period ranged from 12 to 30 months (mean, 18 months).

The mean elbow flexion-extension arc significantly improved from 101 (±22)° to 125 (±12)°. The mean Hand20 score significantly improved from 52 (±22) to 22 (±16). The mean JOA-JES score significantly improved from 67 (±6.5) to 88 (±6.9). The mean MEPS significantly improved from 71 (±12) to 91 (±10). Furthermore, the mean numeric rating scale score for pain significantly reduced from 6.5 (±2) to 2.9 (±1.6) at final follow-up.

There was a significant difference between open and arthroscopic surgery groups only with regard to preoperative JOA-JES scores. Loss of extension and flexion was severe in the open surgery group preoperatively; however, there were no significant differences in Hand20 score, MEPS and pain score between the open and arthroscopic surgery groups both pre- and postoperatively (Table 2).

**Table 2:** Preoperative and postoperative (final follow-up) data

	Preoperative	Postoperative	*P* value
Elbow extension degree (*n* = 11)	−17 (±13)	−7.2 (±7.6)	<.01
Open surgery (*n* = 5)	−22 (±16)	−11 (±8)	n.s.
Arthroscopic surgery (*n* = 6)	−13 (±8.8)	−4.2 (±4.9)	n.s.
Elbow flexion degree (*n* = 11)	119 (±11)	132 (±7.4)	<.01
Open surgery (*n* = 5)	115 (±9)	128 (±8.3)	<.05
Arthroscopic surgery (*n* = 6)	121 (±13)	134 (±5.8)	n.s.
Total arc degree (*n* = 11)	101 (±22)	125 (±12)	<.01
Open surgery (*n* = 5)	93 (±20)	117 (±10)	<.05
Arthroscopic surgery (*n* = 6)	108 (±21)	130 (±9.6)	n.s.
Hand20 score (0–100) (*n* = 11)	52 (±22)	22 (±16)	<.01
Open surgery (*n* = 5)	49 (±23)	19 (±23)	<.01
Arthroscopic surgery (*n* = 6)	53 (±20)	20 (±7.8)	<.05
JOA-JES score (*n* = 11)	67 (±6.5)	88 (±6.9)	<.01
Open surgery (*n* = 5)	63 (±6.1)^a^	88 (±6.4)	<.01
Arthroscopic surgery (*n* = 6)	71 (±4.7)	89 (±8)	<.01
MEPS (*n* = 11)	71 (±12)	91 (±10)	<.01
Open surgery (*n* = 5)	70 (±11)	91 (±13)	<.05
Arthroscopic surgery (*n* = 6)	74 (±14)	93 (±8.2)	<.01
Pain score (0–10) (*n* = 11)	6.5 (±2)	2.9 (±1.6)	<.01
Open surgery (*n* = 5)	6 (±2.8)	2.5 (±2.4)	<.05
Arthroscopic surgery (*n* = 6)	7 (±0.7)	2.6 (±0.5)	<.01

Data are expressed as mean (± standard deviation)

*JOA-JES score* Japanese Orthopaedic Association-Japan Elbow Society Elbow Function Score

*MEPS* Mayo Elbow Performance Score

*n* number

*n.s.* not significant

^a^There was a significant difference between the open and arthroscopic surgery groups (*P* < 0.05)

Measurement of the bony overlap volume showed an intraobserver ICC of 0.93 (95 % confidence interval (CI), 0.76–0.98) for YM, and 0.90 (95 % CI, 0.67–0.97) for MY, and an interobserver ICC of 0.94 (95 % CI, 0.79–0.99).

### Complications

No major complications, including neurovascular compromise or infection, were observed. A postoperative swelling of the elbow due to hematoma was observed in one patient, but did not require surgical drainage and had no adverse effect on the final outcome (case 11).

## Discussion

Clinical outcomes, such as elbow ROM, the numeric rating scale for pain, the Hand20 score, JOA-JES score and MEPS, were significantly improved after computer simulation-assisted elbow debridement arthroplasty.

In this study, the mean flexion-extension arc improved by 24° (101° preoperatively to 125° postoperatively). These results were comparable with the other studies involving the elbow debridement arthroplasty. Miyake et al. reported the results of arthroscopic debridement based on computer simulation of 20 patients, with a mean age of 38 years. The mean flexion-extension arc improved by 23° (98° preoperatively to 121° postoperatively) [7]. Wada et al. described a study of open debridement arthroplasty on 32 patients with a mean age of 50 years. The mean flexion-extension arc improved by 24° (70° preoperatively to 94° postoperatively) [5].

Multi-position CT images can be used to calculate the flexion-extension axis in patients with elbow osteoarthritis [7]. However, multiple CT imaging is not always supported by surgeons and patients in the clinical setting. Although diagnostic X-rays provide great benefits, their use involves some risk of developing cancer. Japan had the highest attributable risks of cancer in the world due to diagnostic X-rays, equivalent to 7587 cases of cancer per year [18].

Recently, Nishiwaki et al. showed that the location of bony impingement in elbow osteoarthritis could be identified by three-dimensional computational modeling [19]. They used the CT data in one position to generate three-dimensional models. Then, they simulated flexion and extension of the elbow by flexing and extending the radius and ulna about the flexion-extension axis. They identified the elbow flexion-extension axis as a line connecting the center of a sphere drawn on the capitellum and a circle drawn on the trochlear groove.

The position and orientation of the elbow flexion axis vary in vivo, which indicates that the elbow flexion-extension axis is not a line, but rather varies throughout the arc of motion. However, the mean axes are located close to a line joining the centers of the trochlea and capitellum [11–14].

A four-dimensional preoperative simulation system was useful for the surgeon because it enabled detection of the impingement lesions pre- and intraoperatively. We used the three-dimensional CT scan data of the affected arm in one position to calculate the anatomical axis. The elbow flexion-extension axis was identified using the “two rings” method in the distal humerus. The “two rings” method was used to calculate the flexion-extension axis of the elbow by converting the surface of the trochlea and capitellum of the humerus into two rings. We evaluated the intraobserver and interobserver reliability of the calculated bony overlap volumes. The intraobserver and interobserver ICCs for calculating the volumes were excellent [15]. This accuracy does not support the entire method, but the method is especially valid for the reproducibility of the flexion–extension axis. Further validation studies may be required to confirm the accuracy of this method.

In the open surgery group, the medial approach was used as the primary approach, and a lateral incision was added when an impingement lesion was present at the radial fossa according to the preoperative simulation. In the arthroscopic surgery group, the surgeon could treat the impingement lesion intensively. Preoperative simulation was useful to help surgeons decide whether a lateral incision was necessary, and where to make an appropriate portal for the arthroscopic shaver.

The present study was based on a preliminary series and it therefore had several limitations, such as a small number of patients. There was no control cohort that would allow a direct comparison of the treatment outcomes with and without this simulation technique. Postoperative CT evaluations were not performed in this series because neither the patients nor the clinicians supported taking postoperative CT scans, even if the clinical symptoms had improved. We did not use a navigation system to identify the impingement lesion intraoperatively by direct inspection, because the navigation system of the elbow is a preliminary technique that has not been well validated. In the future, this problem may be solved by the combination of robotic surgery with a sophisticated navigation system or augmented reality technique [20]. However, the results of this series are encouraging and indicate that four-dimensional preoperative simulation is useful for the identification of impingement lesions and for preoperative decisions regarding the surgical approach. Furthermore, this technique might be useful for other indications such as posttraumatic or rheumatic arthritis of the elbow if we could make two rings on both the trochlea and capitellum of the humerus.

## Conclusion

We showed that four-dimensional, preoperative simulation can be generated by adding the rotation axis to the one-position, three-dimensional CT image of the affected arm. This method can reduce the amounts of radiation exposure and is feasible for elbow debridement arthroplasty.

## Availability of data and materials

The dataset supporting the conclusions of this article is available upon readers request – please contact corresponding author (michi-ya@med.nagoya-u.ac.jp).

## Additional file

Additional file 1: A four-dimensional simulation movie of case 11 is shown. The flexion-extension axis (red line) passes through the centers of the trochlea and capitellum of the humerus. This movie shows the optimal range of motion from 0° to 140° and the impingement area (red lesion) requiring excision. (M4V 2486 kb)

## Abbreviations

CT: computed tomography
CAD: computer-aided design
STL: stereolithography
ROM: range of motion
ICC: intraclass correlation coefficient
JOA-JES score: Japanese Orthopaedic Association-Japan Elbow Society Elbow Function Score
MEPS: Mayo Elbow Performance Score
CI: confidence interval
